# A comparison between microsatellite and single-nucleotide polymorphism markers with respect to two measures of information content

**DOI:** 10.1186/1471-2156-6-S1-S27

**Published:** 2005-12-30

**Authors:** Anbupalam Thalamuthu, Indranil Mukhopadhyay, Amrita Ray, Daniel E Weeks

**Affiliations:** 1 Department of Human Genetics, Graduate School of Public Health, University of Pittsburgh, Pittsburgh, PA 15261, USA; 2 Department of Biostatistics, Graduate School of Public Health, University of Pittsburgh, Pittsburgh, PA 15261, USA

## Abstract

Using the Genetic Analysis Workshop 14 (GAW14) simulated dataset, we compare microsatellite and single-nucleotide polymorphism (SNP) markers in terms of two measures of information content, the traditional entropy-based information content measure, and a new "relative information" measure. Both attempt to measure the amount of information contained in the markers about the identity-by-descent (IBD) sharing among relatives. The performance of the two information measures are compared based on their variability and ability to predict change in the LOD score (ΔLOD) as map density increases for SNP markers. Although in a linked region, LOD scores are correlated with measures of information, we observe that none of the measures predict the LOD score itself very well. In an unlinked region, the LOD score is not related to either measures of information. The information content of microsatellite markers with 7.5-cM spacing is slightly higher than that of SNP markers with 3-cM spacing. At these map densities, microsatellites are found to be uniformly more informative than SNPs irrespective of their level of heterozygosity. For SNPs, we found that as the level of heterozygosity increases, the information content increases. As reported in all other previous studies, we also found that high-density SNPs have higher information content compared to low-density microsatellites. Performance of both the two information measures considered here are similar, but the relative information measure predicts ΔLOD as marker density increases better than the traditional entropy-based information measure.

## Background

Until recently, linkage analysis has been mainly based on widely spaced microsatellite markers (~10 cM), but it is now possible to type very dense single-nucleotide polymorphisms (SNPs) markers at low cost. There has been some debate about whether it is better to use microsatellite or SNP panels. Most comparisons have been made based on the information content (IC) measure proposed by Kruglyak et al. [1]. According to Kruglyak [2], "Information content measures the fraction of inheritance information extracted by the map relative to that which would be extracted by an infinitely dense polymorphic map". If most of the information is extracted by the current map, then it is not necessary to type additional markers. Recently, Nicolae and Kong [3] proposed new measures of relative information (RI) using the allele-sharing exponential statistic of Kong and Cox [4]. In the present article, we consider both these information measures, IC and RI.

Our first objective was to evaluate the IC and RI of microsatellites and SNP markers using the Genetic Analysis Workshop 14 (GAW14) simulated dataset. Using IC, previous studies indicated that high-density SNPs are more informative compared with low-density microsatellites [5-8]. However, these studies have not been done using RI. Also it is of interest to compare microsatellites and SNPs with comparable map densities. Kruglyak [2] found that the density of SNPs with 50% heterozygosity should be approximately 2.5 times that of microsatellites with 75% heterozygosity to have the same information content. For the observed levels of heterozygosity in the GAW14 dataset, based on Kruglyak's results [2], we would expect that the density of SNPs should be approximately 3.33 times that of microsatellites to have the same information content.

Our second objective was to compare the two measures of information, IC and RI, which have only been addressed so far by Nicolae and Kong [3]. Variances were also computed to understand the stability of these two measures. Using a real dataset, Nicolae and Kong [3] observed that RI was uniformly higher than IC. We also expected to see this pattern here.

Finally, we examined changes in IC, RI, and LOD scores as a function of SNP map density. In a linked region, the mean LOD should increase with increasing IC [2]. However, the relationship between mean LOD and RI is unknown. Establishing the relationship between the LOD score and information measures for various map densities should help in determining the appropriate number of additional markers to be typed in a region of interest.

## Methods

The GAW14 simulated data was generated for Kofendred Personality Disorder, a complex heterogeneous disease involving four loci (we obtained the "answers"). Microsatellite and SNPs marker data were available on 10 chromosomes with a SNP map spacing of 3 cM and a microsatellite map spacing of 7.5 cM. We restrict our attention to three groups of nuclear families, (Aipotu, Karangar, and Danacaa groups), with the majority of our results presented only for the Danacaa group. The simulated data consist of 100 replicates for each group of families.

We used ALLEGRO version 1.2 c [9] to calculate IC, RI, and the multipoint allele sharing S_all _LOD scores under the exponential model of Kong and Cox [4] for both SNPs and microsatellites, at each marker position over all 100 replicates. To compare microsatellites and SNPs, we tested the significance of the differences in mean LOD, mean IC, and mean RI by selecting a SNP marker of interest and a microsatellite marker very close to it. We also compared the mean differences between IC and RI for each of the marker types at single marker position of interest. Tests for equality of two means were done using a *t*-test assuming unequal variances. Moreover, to see the pattern of IC and RI as a function of heterozygosity, we grouped the microsatellites (7.5-cM spacing) and SNPs (3-cM spacing) according to their heterozygosity for all 10 chromosomes and calculated the mean IC and mean RI within each group.

To examine the effects of changing SNP density, we purchased 20 high-density SNP packets. In the region surrounding the disease locus on chromosome 1 (as indicated by the "Answers"), we used 6 high-density packets, and 2 each at each end of this chromosome, where there is no disease locus. On chromosome 7, where no disease locus is present, we used another 10 high-density packets. We merged these high-density SNPs with the original data to create a combined dataset with 0.3-cM spacing in these regions. By appropriately dropping markers, we also created regions of SNP markers with approximately 1-cM and 2-cM spacing.

To examine the relationship of LOD scores with IC and RI, we selected the SNP marker C01R0052 (at 167.1 cM) in the disease locus on chromosome 1 and a random locus, C07R0602 (at 97.8 cM) on chromosome 7 for Danacaa group. Let LOD_d_, IC_d _and RI_d _respectively denote LOD, IC and RI of SNPs with map spacing of *d *cM. We tested the significances of correlations (i) *ρ*(Δ*LOD*_*d*1:*d*2_, *IC*_*d*2_) and (ii) *ρ*(Δ*LOD*_*d*1:*d*2_, *RI*_*d*2_), where Δ*LOD*_*d*1:*d*2 _= *LOD*_*d*1 _- *LOD*_*d*2 _with *d*2 >*d*1, and also (iii) *ρ*(*LOD*, *IC*) and (iv)*ρ*(*LOD*, *RI*) at the two loci on the combined 4 SNP maps.

## Results

Using the original data set for microsatellites (7.5-cM spacing) and SNPs (3-cM spacing), we studied all 10 chromosomes for three groups (Danacaa, Aipotu, and Karangar), but most of our results presented here are restricted (due to space) to the Danacca group for chromosome 1 and 7; chromosome 1 contains a strong disease locus, while chromosome 7 contains no disease loci. Detailed results for all three groups are available upon request.

**Figure 1:**
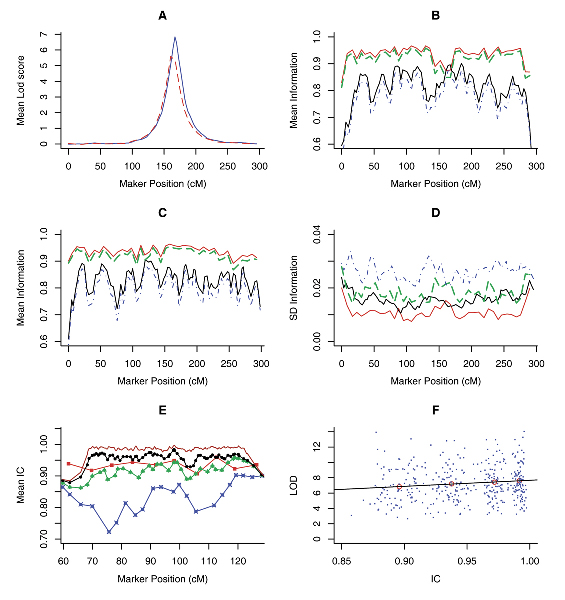
LOD scores, IC and RI for microsatellites and SNPs. A, Mean LOD score for microsatellites (dashed red) and SNPs with 3-cM spacing (solid blue) for chromosome 1. B, Mean IC and RI for microsatellites (IC: upper solid red, RI: long dashed green) and SNPs with 3-cM spacing (IC: lower solid black, RI: dot dashed blue) for chromosome 1. C, Mean IC and RI for microsatellites (IC: upper solid red, RI: long dashed green) and SNPs with 3-cM spacing (IC: lower solid black, RI: dot dashed blue) for chromosome 7. D, Standard deviation of IC and RI for microsatellites (IC: lower solid red, RI: long dashed green) and SNPs with 3-cM spacing (IC: upper solid black, RI: dot dashed blue) for chromosome 1. E, Mean IC for microsatellites (solid Red with squares) and SNPs (0.3 cM: solid Brown, 1 cM: black with dots, 2 cM: green with triangles, 3 cM: blue with stars) for chromosome 7. F, LOD against IC at the disease locus in chromosome 1. Regression line of the scatter plot and average IC at 4 map densities are also shown here. All these figures use marker data for Danacaa group only.

Near the disease locus on chromosome 1, the mean LOD score (Figure 1A) using SNPs (3-cM map) is significantly higher than that obtained using microsatellites (*P*-value ≈ 0.00017 for SNP C01R0052 and microsatellite D01S0023 [at 160.4 cM] in Danacaa group); it is not the case in other regions. The mean IC and mean RI of microsatellites (7.5-cM spacing) are slightly higher than that of SNPs (3-cM spacing) for regions not near the disease locus (Figure 1B and Figure 1C). However, there is an interesting dip in IC and RI of microsatellites near the disease locus on chromosome 1 (Figure 1B), and in this region, IC and RI of SNPs are higher (*P*-values < 10^-13^) than that of microsatellites. Microsatellites (7.5-cM spacing) are uniformly more informative than SNPs (3-cM spacing) at all heterozygosity levels (Table 1). For SNPs, we see that there is a small increase in IC and RI as heterozygosity increases (Table 1). A similar trend is also seen for microsatellites if we ignore the heterozygosity level 0.5–0.6 for three groups.

For microsatellites (7.5-cM spacing) and SNPs (3-cM spacing), mean IC is higher than mean RI throughout all 10 chromosomes for all the groups. The significance of this difference has been examined at randomly selected marker positions. For example, in chromosome 1 in Danacaa group, the *P*-values corresponding to the test of equality of mean IC and mean RI are 4.912 × 10^-8 ^and 3.075 × 10^-22 ^at microsatellite D01S0025 (at 175.1 cM) and SNP C01R0030 (at 94.2 cM), respectively. Variation of RI is higher compared to IC indicating that IC is more stable than RI, as shown in Figure 1D. This pattern is also observed for all the 10 chromosomes in all three groups. For high-density SNPs (0.3-cM spacing), IC and RI are very high and almost equal, and the variance of IC is slightly less than that of RI (data not shown).

Using four different map densities, we observe that, as the map density increases, the SNPs' IC increases uniformly (Figure 1E). Note that IC curve for the 7.5-cM density microsatellite map lies between IC curves for SNPs with map spacing 2 cM and 1 cM (Figure 1E). Similar patterns are observed for the RI curves (not shown here). We find that, in the region shown in Figure 1E, the average heterozygosity of microsatellites and SNPs are 0.76 and 0.35, respectively, and the common allele frequencies vary between 0.501 and 0.98. Therefore, we infer that map density of SNPs must be approximately 3.75 greater than times that of microsatellites to achieve comparable amount of IC, which slightly differs from our expectation based on Kruglyak's [2] study. Note that the end effect in SNPs is more pronounced compared with the microsatellites since the IC curves for SNPs drop down very steeply at the ends of the chromosomes (Figure 1B, C and Figure 1E).

LOD scores and IC are significantly correlated in the linked region ( (LOD, IC) = 0.124, *P*-value 0.013). Figure 1F gives the scatter plot of LOD scores against IC at the SNP marker C01R0052 in chromosome 1 for Danacaa group. Fitted regression line and average LOD score at each map spacing are also shown in Figure 1F. The correlations (i) (Δ *LOD*_2:3_,*IC*_3_) = -0.237 (ii) (Δ *LOD*_1:3_,*IC*_3_) = -0.256, (iii) (Δ *LOD*_0.3:3_,*IC*_3_) = -0.233 are significant (*P*-values < 0.019). The correlations of ΔLOD with RI are higher (-0.405, -0.342, -0.278, respectively). These correlations reveal the difference in LOD score from sparsely spaced SNPs to high-density SNPs can be assessed through the IC or RI values of the low density SNPs. Although IC is significantly correlated with LOD, Figure 1F shows that individual LOD scores may not be predicted well by IC, because of large variability in LOD scores within a small region of IC values (similar results are obtained for RI). The scatter plots of Δ*LOD*_*d*1:*d*2 _with IC_*d*2 _also give the same pattern as Figure 1F. The correlation between LOD and IC in an unlinked region (on chromosome 7 at SNP C07R0602) is not significant. Therefore, in both the linked and unlinked regions, none of the measures would predict the LOD score well.

## Discussion

We compared information contained in microsatellites to that of SNPs using the GAW14 simulated dataset. Based on the level of heterozygosity for SNPs and microsatellites in our dataset, the ratio (~3.75) of map densities of SNPs to microsatellites, to have same information content, is slightly higher than that predicted (~3.3) by the results of Kruglyak [2]. In his study, Kruglyak [2] used only 10 cousin pairs with 100 replicates, whereas we have used simulated data containing 100 nuclear families in each of 100 replicates in three different populations. Therefore, our results provide more general conclusions about the relative information content of microsatellites and SNPs. However, it is interesting to note that at the close proximity of disease locus in chromosome 1, IC for SNPs is significantly higher than that of microsatellites. This may be due to the fact that the microsatellite markers are slightly away from the disease locus, while there is a SNP marker inside the disease locus. We could not study this pattern of dip near the other disease loci, since the other disease loci were located at the extreme ends of chromosomes. We also observe that with the increase in heterozygosity for SNPs, there is slight increase in IC and RI. The results by Evans and Cardon [5] show that IC for microsatellites increases as heterozygosity increases. But this pattern is not as clear in our study. This may be due to the fact that they calculated the IC at the middle of the interval between two markers using equally frequent alleles for their microsatellites.

In contrast to Nicolae and Kong's [3] results, we find that IC is uniformly higher than RI for microsatellites as well as for SNPs. Moreover, the variance of RI is uniformly greater than that of IC, indicating that IC is a more stable measure of information. However, the correlations of RI with Δ*LOD*_*d*1:*d*2 _are slightly higher than those of IC, indicating that RI is more closely related to the increase in the LOD scores when the map density of SNPs is increased. We also observe that neither IC nor RI is able to predict LOD scores very well.

For high-density SNPs (0.3-cM spacing), the IC and the RI are very high. Based on our study, the SNP spacing must be less than 2 cM on average to have comparable IC to that of microsatellies with a map spacing of 7.5 cM. In this study, the effect of linkage disequilibrium on information measures has not been examined because there is no linkage disequilibrium in the regions in which we purchased high density SNPs. It would be interesting to examine this in a future study. Also it is evident from previous studies and our present study that study design plays an important role in determining the appropriate map density for SNPs to obtain the same amount of information as given by microsatellites.

## Abbreviations

GAW: Genetic Analysis Workshop

IBD: Identity by descent

IC: Information content

RI: Relative information

SNP: Single-nucleotide polymorphism

## Authors' contributions

DEW designed and coordinated the study and helped draft the manuscript. AT and IM contributed equally to analyses and draft of the manuscript. AR helped in statistical analysis. All the authors read and approved the final manuscript.

## Figures and Tables

**Table 1:** Mean IC and RI for microsatellite and SNP markers with respect to heterozygosity.

		Heterozygosity									
		
Microsatellite (7.5-cM spacing)								SNP (3-cM spacing)			
	
Group		0.4–0.5	0.5–0.6	0.6–0.7	0.7–0.8	0.8–0.9	0–0.1	0.1–0.2	0.2–0.3	0.3–0.4	0.4–0.5
Danacaa	IC	0.847	0.891	0.884	0.895	0.898	0.805	0.814	0.817	0.823	0.825
	RI	0.808	0.865	0.856	0.870	0.874	0.783	0.783	0.788	0.794	0.796
Karangar	IC	0.850	0.891	0.884	0.895	0.898	0.805	0.813	0.817	0.823	0.824
	RI	0.812	0.865	0.855	0.870	0.874	0.774	0.783	0.787	0.793	0.795
Aipotu	IC	0.845	0.878	0.884	0.894	0.897	0.806	0.814	0.817	0.822	0.824
	RI	0.810	0.854	0.857	0.870	0.873	0.776	0.785	0.788	0.794	0.796
